# TLR2 Ligands Induce NF-κB Activation from Endosomal Compartments of Human Monocytes

**DOI:** 10.1371/journal.pone.0080743

**Published:** 2013-12-12

**Authors:** Karim J. Brandt, Céline Fickentscher, Egbert. K. O. Kruithof, Philippe de Moerloose

**Affiliations:** Division of Angiology and Hemostasis, University Hospital of Geneva and Faculty of Medicine, Geneva, Switzerland; McGill University, Canada

## Abstract

Localization of Toll-like receptors (TLR) in subcellular organelles is a major strategy to regulate innate immune responses. While TLR4, a cell-surface receptor, signals from both the plasma membrane and endosomal compartments, less is known about the functional role of endosomal trafficking upon TLR2 signaling. Here we show that the bacterial TLR2 ligands Pam_3_CSK_4_ and LTA activate NF-κB-dependent signaling from endosomal compartments in human monocytes and in a NF-κB sensitive reporter cell line, despite the expression of TLR2 at the cell surface. Further analyses indicate that TLR2-induced NF-κB activation is controlled by a clathrin/dynamin-dependent endocytosis mechanism, in which CD14 serves as an important upstream regulator. These findings establish that internalization of cell-surface TLR2 into endosomal compartments is required for NF-κB activation. These observations further demonstrate the need of endocytosis in the activation and regulation of TLR2-dependent signaling pathways.

## Introduction

The innate immune system provides a first line of defense against various pathogens without the requirement of prior exposure to foreign antigens. Among the members of the pattern-recognition receptors, Toll-like receptors (TLR) play a central role in innate immunity. A fundamental principle that governs all aspects of TLR signal transduction is that the mechanisms that ensure the fidelity of signaling are determined by their cellular localization and selective regulators of TLR signal transduction [1]. Endocytosis of plasma membrane–localized TLRs was initially thought to attenuate ligand-induced responses, but it is now widely accepted that receptor internalization permits both the propagation of the signaling cascade from endosomal compartments and the generation of distinct signaling events [2-4]. Although less is known about the regulators that control TLR endocytosis after microbial detection, new accumulating evidence indicates that accessory proteins, such as CD14, are not only critical in ligand recognition but may also fulfill additional functions [5]. TLR2 recognizes various ligands, and makes use of different mechanisms to provide specificity to each of them. In this context, recognition by receptor heterodimerization and/or endocytosis gives rise to broader ligand specificity [3,6,7]. In particular, by association with TLR1 and/ or TLR6, TLR2 is able to recognize bacterial triacylated or diacylated lipopeptides [8]. Indeed, the role of TLR1 and TLR6, as well as that of CD14, in cell activation in response to lipoteichoic acid (LTA) and the synthetic bacterial lipopeptide Pam3-Cys-Ser-Lys4 (Pam_3_CSK_4_) is well established [7,9]. Like TLR4, TLR2 has been demonstrated to associate with CD14 to increase cellular responses to ligands [7,10,11]. CD14 regulates TLR4 endocytosis and hence the subsequent endosomal-dependent signaling pathway [5]. In addition to the TLR4 signaling that takes place at the plasma membrane through MyD88/TIRAP adaptor proteins leading to early NF-κB activation, a second signaling event was demonstrated to be initiated from the endosomal compartments through TRAM/TRIF adaptor proteins mediating late NF-κB signaling and phosphorylation of the transcription factor Interferon Regulatory Factor-3 (IRF3), which in turn regulates type I interferon (IFN) [12]. While an intracellular localization of TLR2 has been observed [3,13,14], its relevance remains to be clearly established in TLR2 signaling. Indeed, Nilsen et al. have shown that a dominant negative form of Dynamin had no effect on TLR2 signaling whereas several other studies have shown that production of TNF and IL-6 is altered by inhibition of TLR2 internalization [11,15].

In this study, we investigated the mechanism mediating the activation of NF-κB in TLR2-activated human monocytes. We demonstrate that NF-κB activation in primary monocytes in response to LTA and Pam_3_CSK_4_ requires internalization of TLR2 through clathrin-coated pits, and that this mechanism is regulated by CD14.

## Materials and Methods

### Ethics statement

Buffy coats of blood of healthy donors were provided by the Geneva Hospital Blood Transfusion Center. In accordance with the ethical committee of the Geneva Hospital and with the Declaration of Helsinki, the blood bank obtained informed consent from the donors, who are thus informed that part of their blood will be used for research purposes.

### Reagents

Ultra-pure LPS K12, LPS conjugated-biotin; lipoteichoic acid (LTA) from *Staphylococcus aureus*, Pam_3_CSK_4_ conjugated-biotin, blocking peptide against MyD88 and TRIF were from InvivoGen (San Diego, CA). Pam_3_CSK_4_ was from Alexis Corporation (San Diego, CA). IFNγ was from Laboraoire Roussel et Cie, SNC, Paris. Chloroquine diphosphate, Chlorpromazine hydrochloride, Dynasore and Ammonium chloride are from Sigma (Sigma, St. Louis, MO). Monoclonal anti-human CD14 blocking antibody, AF488-conjugated mouse anti-human TLR2 and TLR4, anti-human CD32 antibodies were from Biolegend. Secondary antibodies were from Jackson ImmunoResearch Laboratories, Inc (West Grove, PA). 

### Cell culture

Monocytes were isolated from blood buffy coats of healthy volunteers as previously described [16]. Monocyte purity routinely consisted of >90% CD14^+^ cells, <1% CD3^+^ cells, and <1% CD19^+^ cells as assessed by flow cytometry. Cells were cultured in RPMI containing 10% Fetal Bovine Serum (FBS; Gibco BRL-Life Technologies).

Human embryonic kidney 293 (HEK293) cells stably transfected with human TLR4, MD2 and CD14 (HEK-Blue4™) or with human TLR2 and CD14 (HEK-Blue2™) were obtained from InvivoGen (San Diego, CA) and grown in Dulbecco’s Modified Eagle’s Medium containing 10% FBS. The HEK-Blue cells express the secreted embryonic alkaline phosphatase reporter genes (SEAP) under the control of promoter containing five NF-κB binding sites, which enables to quantify cell activation by measuring SEAP activity in media containing specific enzyme substrate. HEK-293 cells expressing only TLR2 were obtained as previously described [17].

### TNF production

Blocking antibodies and peptides were used at 10μg/ml and 50μM or pharmacological inhibitors were added prior to incubation for 24h with 1μg/ml LTA, 100ng/ml Pam_3_CSK_4_ or IFNγ (500U/ml). Culture supernatants were tested for the production of TNF by a commercially available enzyme immunoassay (R&D Systems, Minneapolis, MN, USA). 

### mRNA Silencing

HEK-Blue2™ and HEK-Blue4™ were transfected for 72h with 100nM of Stealth siRNA against heavy chain clathrin 17 (CHC17) designed by the supplier (Life Technologies, Grand Island, NY) or with 100nM of negative control duplex. Transfections were done using TransIT-TKO, according to the supplier's protocol (Mirus, Madison, WI). CHC17 silencing was ascertained by Western blot. Production of SEAP enzyme by silenced HEK-Blue2™ was measured with HEK-Blue™ Detection Medium (InvivoGen, San Diego, CA). Cell’s activation was assessed by measuring the absorbance at 650 nm.

### Beads assay

200ng/ml of a solution of LTA conjugated-biotin, Pam_3_CSK_4_-biotin or LPS-biotin were bound to Streptavidin-Agarose Resin (Thermo Fisher). After ligand binding, the supernatants were conserved and the beads washed 3 times. HEK-Blue2™ cells or HEK-Blue4™ cells were treated with soluble LTA-biotin (200ng), Pam_3_CSK_4_-biotin or LPS-biotin (20ng), with beads-bound ligands at corresponding quantities or with the supernatants remaining after incubation of biotin-conjugated ligands with Streptavidin-Agarose. The capacity of ligand treated HEK-Blue2™ or HEK-Blue4™ cells to produce SEAP enzyme was measured with HEK-Blue™ Detection Medium. Cells activation was assessed by measuring the absorbance at 650 nm. The ability of the conjugated-beads to interact with respective receptors is evaluated by flow cytometry. Briefly, HEK-Blue2™ cells or HEK-Blue4™ cells were treated with beads-bound ligands for 1h. Beads were isolated and washed 3 times with PBS then HEK-Blue2™ cells or HEK-Blue4™ cells associated to the beads through interaction between ligand and receptors are quantified using antibodies against TLR2 and LTR4 by flow cytometry.

### Real-time quantitative PCR

mRNA was prepared by TriReagent^®^ (Molecular Research Center) according to the provided protocol. qPCR was done on a StepOne™ instrument (Life Technologies, Grand Island, NY). TNF and IFN-β TaqMan probes and master mix were from Life Technologies, Grand Island, NY. Targets mRNA expression were normalized against the expression of 18S ribosomal mRNA analyzed simultaneously. Data were analyzed using the comparative ΔC_T_ method. 

### Western blot

Total cell lysate was prepared and subjected to Western blot analysis as described previously [16]. The blots were probed with: anti-IκB and anti-phospho-IRF3 (Cell Signaling, Danvers, MA), anti-CD14 (Santa Cruz, Dallas) and anti-β-tubulin (Sigma, St. Louis, MO). Secondary antibodies conjugated to IR700 and IR800 (Rockland, Gilbertsville, PA) were used. Antibody-bound proteins were detected and quantified by the Odyssey system (Li-Cor, Lincoln, NE).

### Flow cytometry

For staining of extracellular proteins, cells were stained with mouse anti-biotin/ goat anti-mouse-phycoerythrin (Jackson, West Grove, PA). For intracellular staining, cells were activated as described above, trypsinized, fixed and permeabilized. Staining was assessed with ACCURI C6 flow cytometer (BD Biosciences, San Jose, CA).

### Statistical analysis

Where indicated, significance of differences between groups was assessed using Student’s paired t test. *:p ≤ 0.05; **:p ≤ 0.005; ***:p ≤ 0.0005. All data are represented as mean +/- SD of at least 3 independent experiments.

## Results

### Clathrin-dependent endocytosis controls TNF expression induced by LTA and Pam_3_CSK_4_

**Figure 1:**
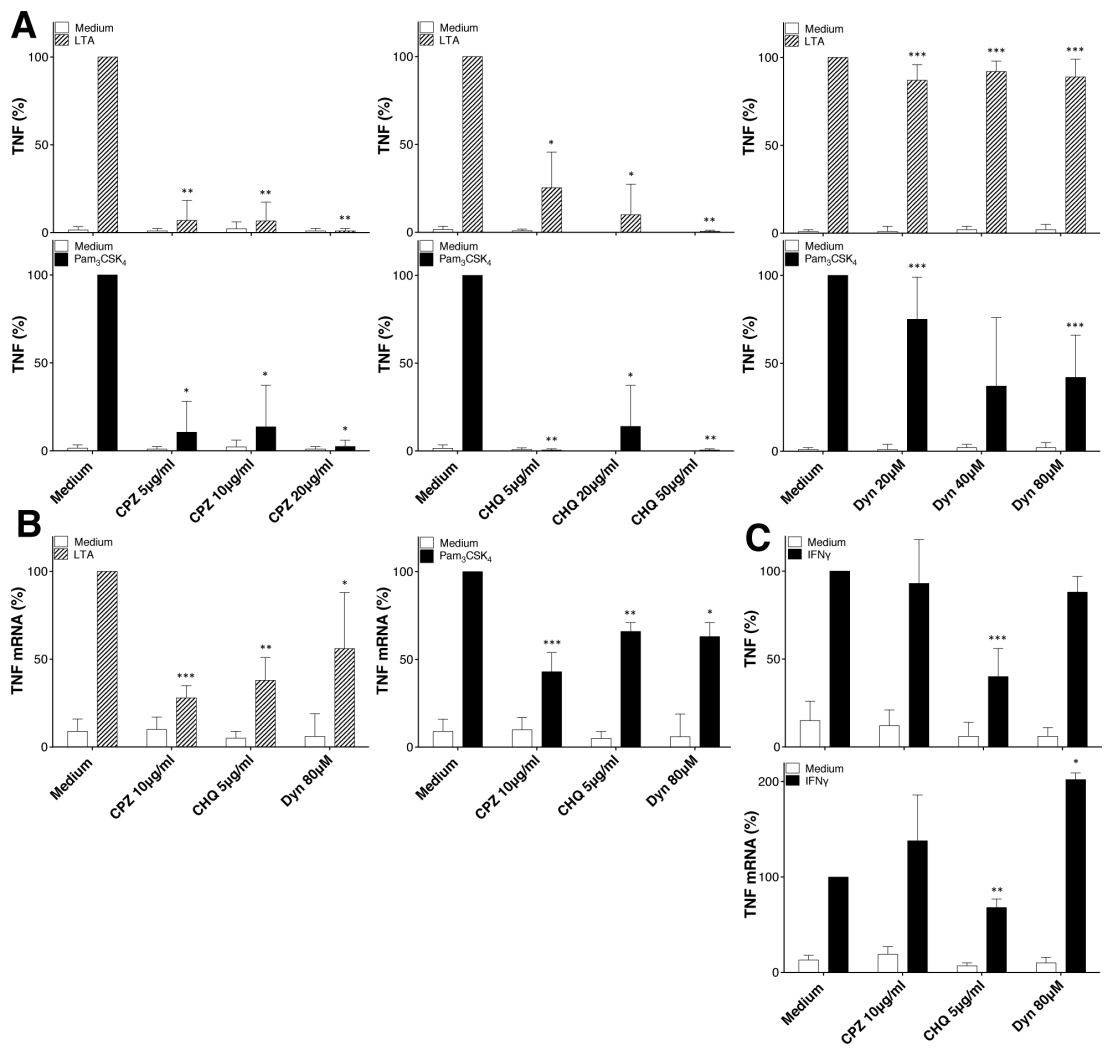
Effect of endocytosis inhibitors on TLR2 mediated induction of TNF in primary human monocytes. Monocytes were treated with pharmacological endocytosis inhibitors during 45min prior to treatment for 4h or 24h with LTA (1μg/ml) and Pam_3_CSK_4_ (100ng/ml). (**A**) Dose response of the effect of chlorpromazine (CPZ), chloroquine (CHQ) and Dynasore (Dyn) on TNF secretion in TLR2 ligand-activated monocytes. TNF response to LTA and Pam_3_CSK_4_ after 24h is strongly reduced by all three endosomal pathway inhibitors. (**B**) Effect of CPZ, CHQ and Dyn on TNF mRNA expression in LTA- and Pam_3_CSK_4_-activated monocytes at 4h. TNF mRNA response to LTA and Pam_3_CSK_4_ is reduced by all three endosomal pathway inhibitors. (**C**) Effect of CPZ, CHQ and Dyn on TNF mRNA and TNF production after 4h and 24h, respectively, in IFNγ-activated monocytes. Data were normalized to the TNF production observed in the absence of inhibitors. For all panels data are represented as mean +/- SD of at least 3 independent experiments. *:p ≤ 0.05; ** p ≤ 0.005; *** p ≤ 0.0005.

Recent data indicate a key role for the internalization of TLR4 and TLR2 molecules expressed at the plasma membrane by a process dependent on clathrin and the GTPase dynamin in the triggering of specific signals from early endosomes [2,18]. The previously described localization of TLR2 ligands within endosomal compartments [18,19] suggests a possible role of endocytic pathways in TLR2 signaling. To assess the role of TLR2 internalization in the induction of TNF in monocytes, cells were treated with different inhibitors: Chlorpromazine (CPZ), an inhibitor of clathrin-dependent endocytosis; Chloroquine (CHQ), an inhibitor of endosomal maturation; and Dynasore (Dyn), a specific inhibitor of dynamin which is crucial for pinching off of clathrin-coated pits and other vesicular trafficking processes. In addition, we used a non-specific inhibitor of vesicular acidification: NH_4_Cl. This weak base inhibits endosomes maturation by increasing the vesicular pH [20,21]. As shown in Figure 1 and Figure S1A, CPZ, CHQ and Dyn significantly decreased the expression of TNF in LTA- and Pam_3_CSK_4_-activated monocytes (Figures 1A-B and S1A). The effects of CPZ, CHQ, Dyn and NH_4_Cl were not due to direct cytotoxic effects of the drugs on monocytes (Figure S1B), nor to a non-specific inhibition of cellular responses, because these drugs had no effect on the TNF expression in IFNγ-activated monocytes (Figure 1C) meaning that translation and release of TNF are not affected by endocytosis inhibitors. Our results suggest that internalization of TLR2 is required to increase TNF expression in LTA- and Pam_3_CSK_4_-activated monocytes. The mean ± SD of all experiments are presented in Table 1. The variations were due to difference in response of the different monocytes preparations used. 

**Table 1:** 1TNF production by isolated human monocytes.

**Stimulus**	**TNF (pg/ml)**
Medium	0.6 ± 1.5
LTA (1 µg/ml)	4956 ± 2709
Pam_3_CSK_4_ (100 ng/ml)	1573 ± 808
LPS (100 ng/ml)	8071 ± 3664

Due to the great variation of TNF and TF mediator production among the different monocyte preparations (blood donors) used in this study, data are expressed as mean ± SD of all experiments. We therefore present all results as percentages of TNF production observed in the absence of inhibitors.

### Clathrin mediated endocytosis controls NF-κB activation by LTA and Pam_3_CSK_4_

**Figure 2:**
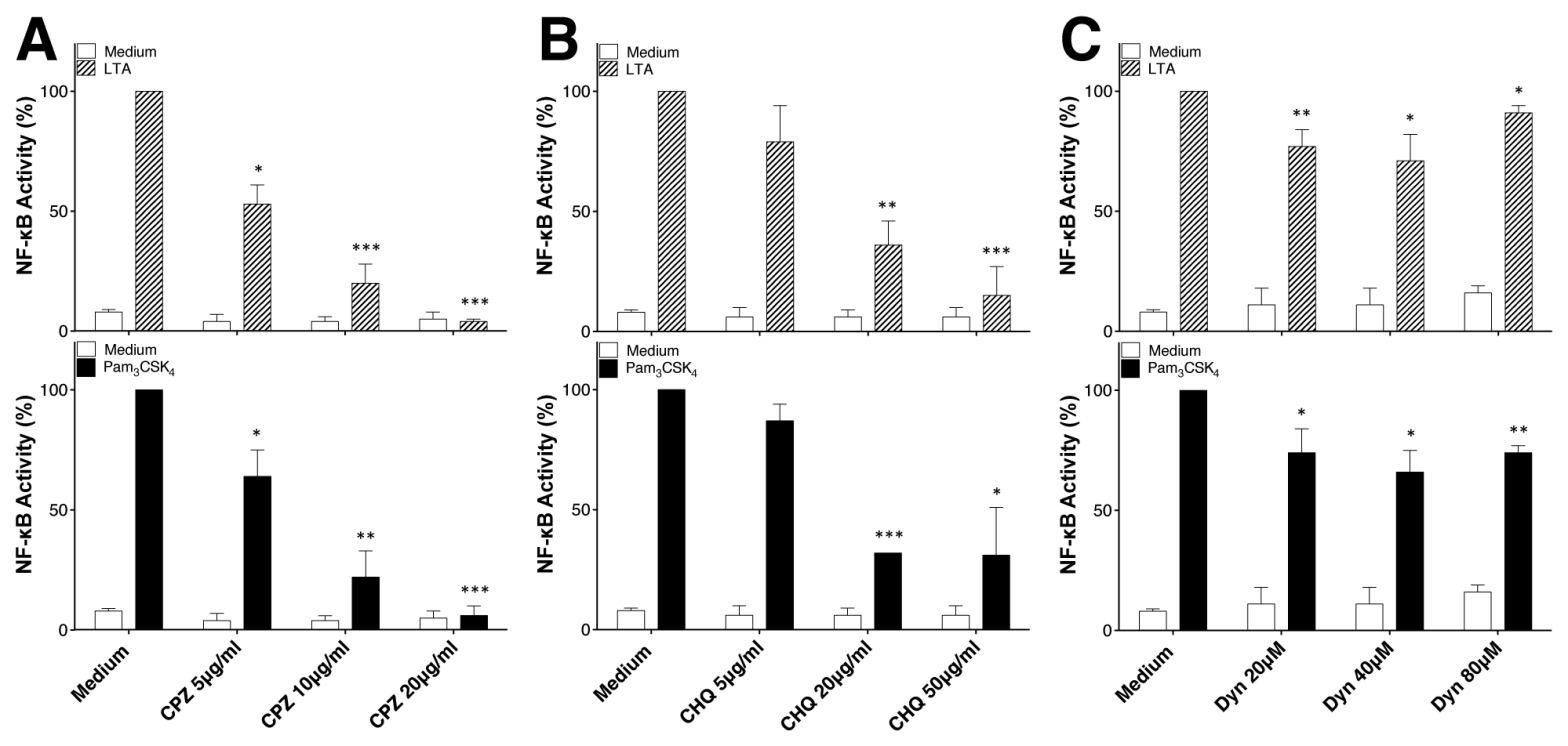
Effect of endocytosis inhibitors on TLR2 mediated induction of NF-κB in HEK-Blue2™ cells. (**A-C**) Dose response of the effect of CPZ, CHQ and Dyn on NF-κB activity in LTA- and Pam3CSK4-activated HEK-Blue2™ cells, which express soluble alkaline phosphatase (SEAP) under control of a promoter containing five NF-κB binding sites. LTA- and Pam3CSK4-induced NF-κB activity was reduced by all endocytic pathway inhibitors. NF-κB activity was monitored in cells supernatant by SEAP enzyme activity measured with HEK-Blue™ Detection Medium, which contains a specific SEAP substrate. Data are represented as mean +/- SD of at least 3 independent experiments. *:p ≤ 0.05; **:p ≤ 0.005; ***:p ≤ 0.0005

Taking in consideration the primary role of NF-κB in regulating monocytic TNF expression in response to LPS [22,23] and the data in Figure 1 suggesting a key function of endocytosis in favoring TNF production by monocytes, we hypothesized that TLR2 internalization is required for NF-κB activation. To this end, we took advantage of HEK-Blue2™ cells which express TLR2, CD14 and a secreted embryonic alkaline phosphatase (SEAP) reporter gene under the control of a promoter containing five NF-κB binding sites. As shown in Figures 2A-C and S1C, the treatment of HEK-Blue2™ cells with CPZ, CHQ, Dyn and NH_4_Cl decreased the activation of NF-κB in response to TLR2 ligands and subsequent secretion of SEAP.

**Figure 3:**
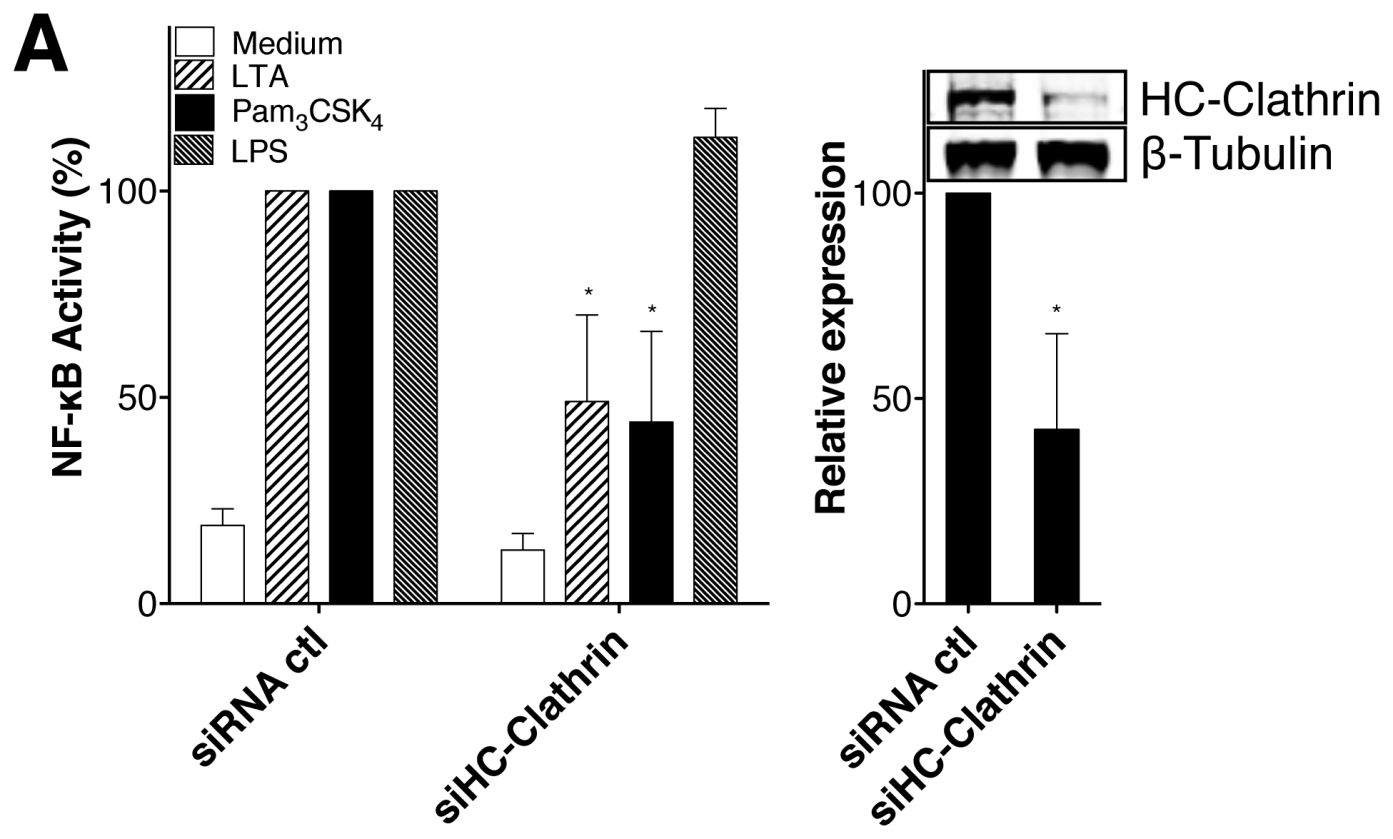
Effects of clathrin knockdown on NF-κB responses to LTA and Pam_3_CSK_4_. (A) The right panel shows quantification by western blot of the heavy chain of clathrin in HEK-Blue2™ and representative experience obtains with HEK-Blue2™ and HEK-Blue4™cells treated with stealth siRNA for the heavy chain of clathrin or Stealth RNAi™ negative control duplex. The left panel shows NF-κB activity of HEK-Blue2™ and HEK-Blue4™cells siRNA-treated for 72h and then activated with Pam3CSK4, LTA or LPS (100ng/ml, 1μg/ml, 100ng/ml, respectively) for 24h. Cells were tested for NF-κB activity by measuring SEAP activity in cell supernatants. NF-κB activity is presented as percentage of production in mock-siRNA transfected HEK-Blue2™or HEK-Blue4™ cells. Data are represented as mean +/- SD of at least 3 independent experiments.

As an alternative approach to the use of pharmacological inhibitors, we analyzed the functional consequences of gene silencing of the clathrin heavy chain (HC-Clathrin) on TLR2 ligand-induced NF-κB activation. As shown in Figure 3A, left panel, the silencing of HC-Clathrin expression in HEK-Blue2™ cells decreased NF-κB activity induced by LTA and Pam_3_CSK_4_ confirming the results obtained with CPZ (Figures 1A and 2A). Furthermore, the silencing of Clathrin expression in HEK-Blue4™ cells does not affect significantly NF-κB activity induced by LPS (Figure 3A). The partial inhibition by clathrin siRNA of NF-κB activity is consistent with the partial silencing of the clathrin heavy chain, as assessed by immunoblot quantification (Figure 3A, right panel). Our results indicate that the clathrin-dependent internalization of TLR molecules expressed at the cell surface (i.e. TLR1/2/6) lead to NF-κB activation by their respective ligands.

### LTA and Pam_3_CSK_4_ induce NF-κB activation from endosomal compartments

**Figure 4:**
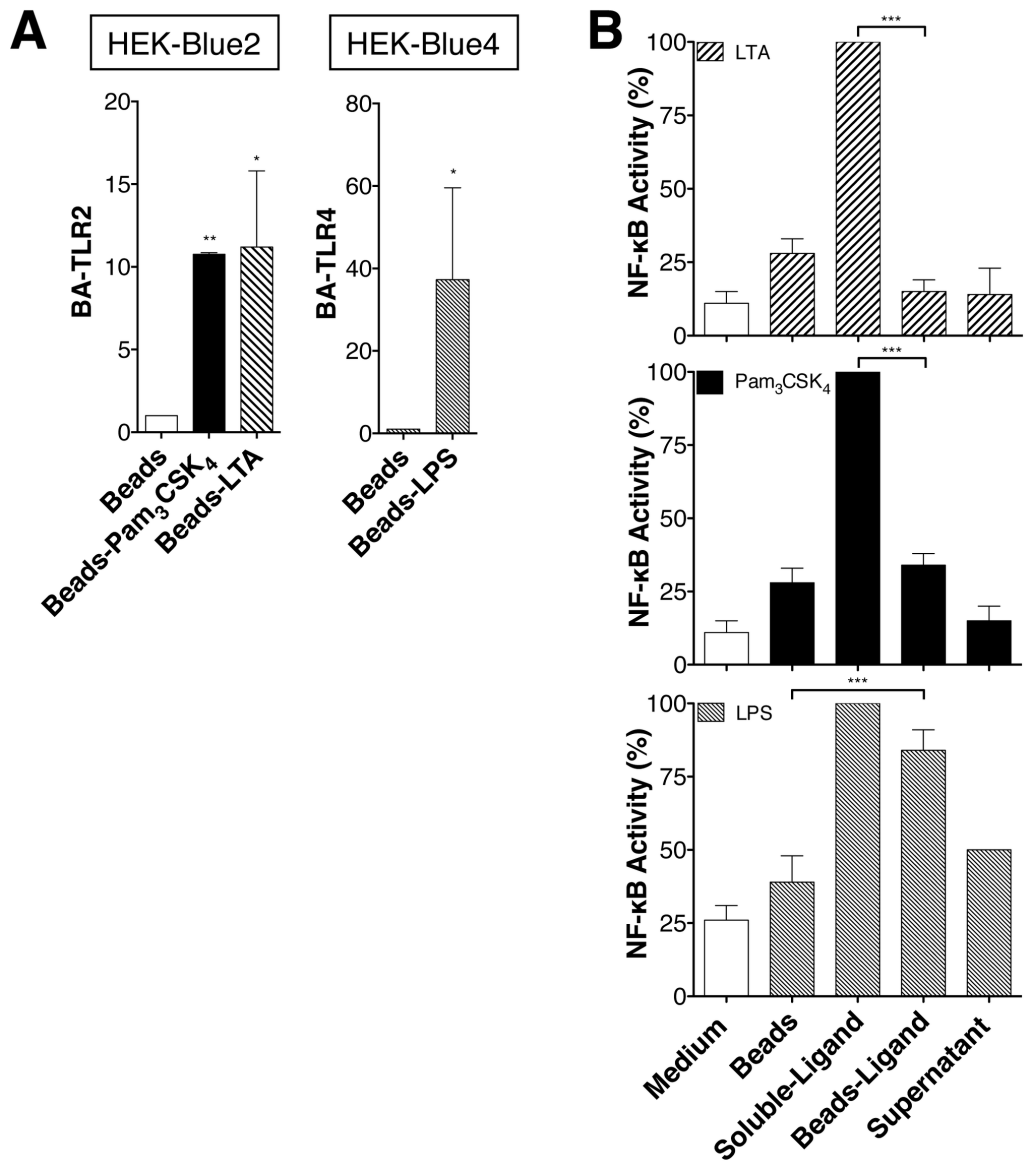
LTA and Pam_3_CSK_4_ internalization allows NF-κB activation. (**A**) HEK-Blue2™ or HEK-Blue4™ cells were treated with LTA-biotin, Pam_3_CSK_4_-biotin, LPS-biotin beads-bound ligands as well as Streptavidin-beads. After beads isolation, the amount of beads-associated HEK-Blue2™ (BA-TLR2) and HEK-Blue4™ (BA-TLR4) cells was measured by flow cytometry using anti-TLR2 and anti-TLR4 antibodies. Results are expressed as the ratio of the geometric mean of TLR2 or TLR4 fluorescence (GMEAN) ± SD of three experiments. Final GMEAN values are the result of GMEAN subtraction from isotype control. (**B**) HEK-Blue2™ or HEK-Blue4™ cells for LPS, were treated with soluble LTA-biotin (1μg/ml), Pam_3_CSK_4_-biotin (100ng/ml), LPS-biotin (100ng/ml) or LTA-biotin, Pam_3_CSK_4_-biotin, LPS-biotin beads-bound ligands (containing the same concentrations of TLR ligands as the soluble forms) and ligands depleted supernatants for 24h. NF-κB activity is monitored by SEAP enzyme activity measured with HEK-Blue™ Detection Medium, which contains a specific SEAP substrate. Data are represented as mean +/- SD of at least 3 independent experiments.

To determine whether the internalization is important for NF-κB activation, Pam_3_CSK_4_-conjugated biotin and LTA-conjugated biotin were fixed to Streptavidin-coated beads (bead size 50-80 μm). The size of the beads prevents their endocytosis but does not interfere with binding of LTA and Pam_3_CSK_4_ or LPS to TLR2 and TLR4 [15,19]. Indeed, HEK-Blue2™ cells are associated to LTA- and Pam_3_CSK_4_-coated beads as similar manner than HEK-Blue4™ cells to LPS-coated beads (Figure 4A). When bound to the beads, LTA and Pam_3_CSK_4_ were unable to induce NF-κB activation in HEK-Blue2™ cells (Figure 4B). In contrast, NF-κB activation in HEK-Blue4™ cells induced by LPS (a TLR4 ligand) which is known to activate NF-κB from the plasma membrane and endosomal compartment, was hardly affected when fixed to the beads (Figure 4B, third panel).

**Figure 5:**
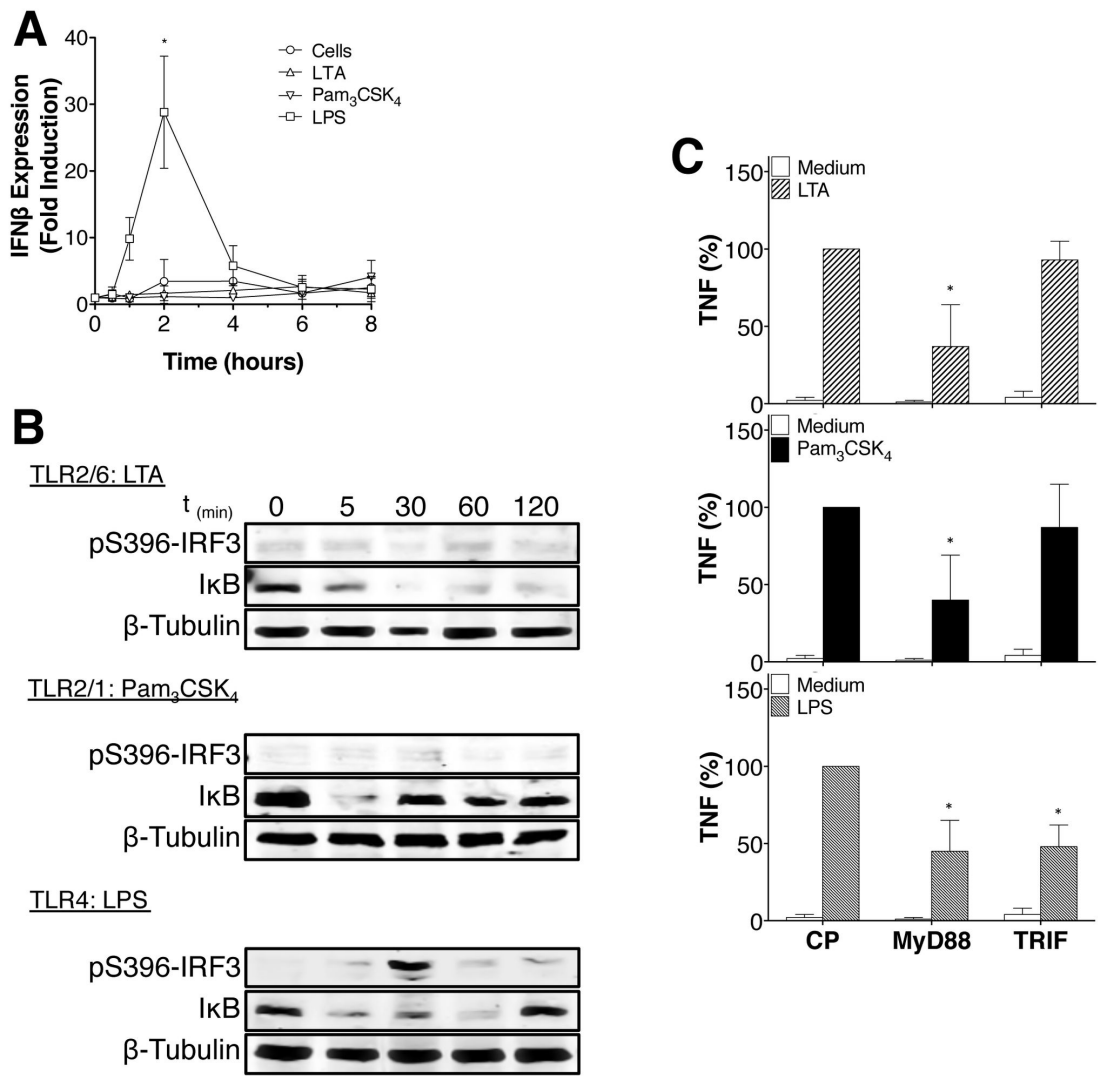
LTA and Pam_3_CSK_4_ do not induce IRF-3 phosphorylation or IFN-β production. (**A**) Human monocytes were treated with LTA, Pam_3_CSK_4_, or LPS (1μg/ml, 100ng/ml and 100ng/ml respectively) for the indicated time periods. IFN-β expression was assayed by quantitative PCR. Data are represented as mean +/- SD of results obtained with monocytes from at least 3 different blood donors. (**B**) Human monocytes were treated with LTA, Pam_3_CSK_4_ or LPS (1μg/ml, 100ng/ml and 100ng/ml respectively) for indicated time and the degradation of IκB (NF-κB) and phosphorylation of IRF3 analyzed by Western blot. Data are representative of 3 independent experiments. (**C**) Human monocytes were treated with 50μM of blocking peptides for MyD88 and TRIF or control peptide (CP) during 60min prior to be activated for 24h with LTA (1μg/ml), Pam_3_CSK_4_ (100ng/ml) or LPS (100ng/ml). MyD88 blocking peptide reduces TNF response to LTA and Pam_3_CSK_4_ after 24h. Data are represented as mean +/- SD of results obtained with monocytes from at least 3 different blood donors.

Data indicate that vaccinia virus and bacterial ligands are not only able to produce type I interferon through TLR2 and IRF family members but are also capable of activating NF-κB [3,24]. In contrast to studies suggesting that TLR2 can activate IFN-β from endosomal compartments and pro-inflammatory cytokines from the plasma membrane [24], our data indicate no IRF-3 phosphorylation or IFN-β production in LTA- or Pam_3_CSK_4_-activated monocytes. However, as previously described, LPS induced IRF-3 phosphorylation, IFN-β production and NF-κB activation (Figure 5A-B). In accordance with previous results [25], MyD88 and TRIF are required for TNF production by LPS-treated monocytes from plasma membrane or endosome, respectively, but MyD88 only is required for TNF production induced by LTA and Pam_3_CSK_4_ in monocytes (Figure 5C and Figure S2). These results suggest that although present at the plasma membrane, the localization of TLR2 within an endosomal compartment is required to trigger NF-κB activation in response to Pam_3_CSK_4_ or LTA. This additional mechanism is not functionally coupled to the triggering of an IRF3/ IFNβ response. In conclusion, while ligands bind to TLR2 at the cell surface, they initiate NF-κB activation from endosomal vesicles by a MyD88-dependent mechanism.

### CD14 controls internalization and NF-κB activation in LTA- and Pam_3_CSK_4_-activated cells

**Figure 6:**
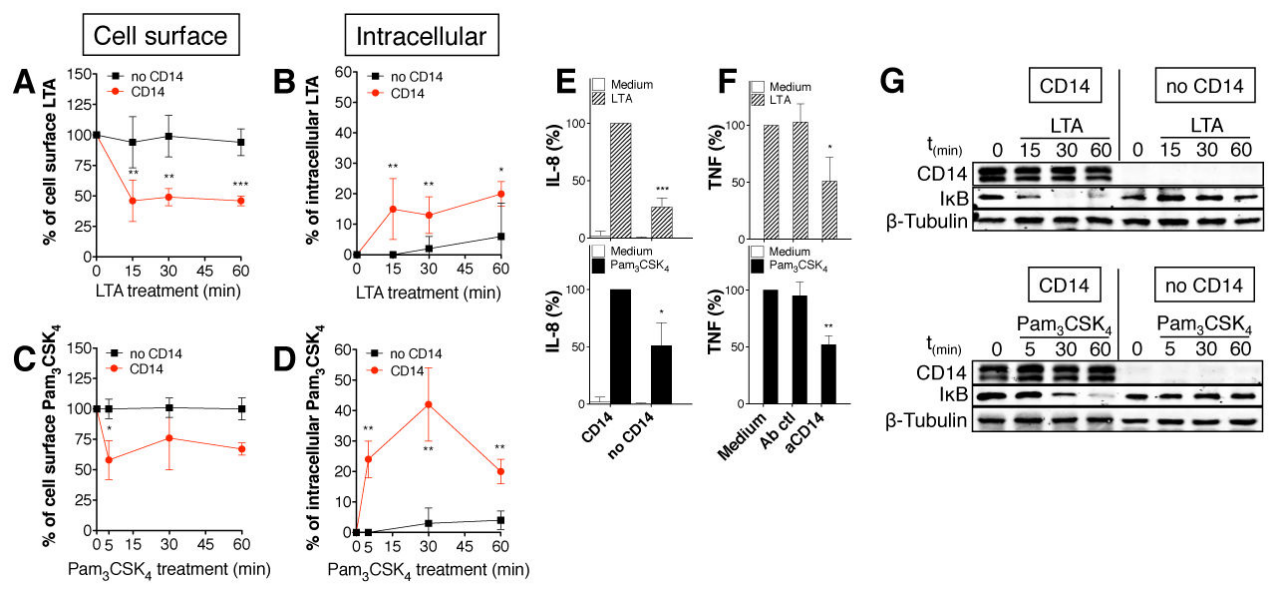
CD14 controls LTA and Pam_3_CSK_4_ internalization and NF-κB activation. HEK-Blue2™ cells are TLR2^+^ and CD14^+^ (CD14) while HEK-TLR2 cells are TLR2^+^ and CD14^-^ cells (no CD14). (**A** and **C**) HEK-Blue2™ cells ((red dot) and HEK-TLR2 cells (■ were treated with LTA-biotin 1μg/ml or Pam_3_CSK_4_-biotin 100ng/ml and TLR2 ligands endocytosis was measured by flow cytometry at the times indicated. Displayed are the mean +/- SD of the percentage of corrected fluorescence index (MFI) of specific extracellular TLR2 ligands staining at each time point. (**B** and **D**) HEK-Blue2™ cells ((red dot) and HEK-TLR2 cells (■ were treated with LTA-biotin 1μg/ml or Pam_3_CSK_4_-biotin 100ng/ml and intracellular accumulation of TLR2 ligands was measured by flow cytometry at the times indicated. Displayed are the mean +/- SD of the percentage of corrected fluorescence index (MFI) of specific intracellular TLR2 ligands staining at each time point. (**E**) HEK-Blue2™ cells and HEK-TLR2 cells were activated by indicated TLR2 ligands (LTA 1μg/ml or Pam_3_CSK_4_ 100ng/ml) and IL-8 production was assayed by ELISA. Data are represented as mean +/- SD of at least 5 independent experiments. (**F**) Monocytes were treated with blocking antibody against CD14 (10μg/ml) during 45min prior to be activated with Pam_3_CSK_4_ 100ng/ml or LTA 1μg/ml. After 24h, TNF secretion was assayed by ELISA. CD14 blocking antibody decreases significantly TNF production. Data are represented as mean +/- SD of at least 4 independent experiments. (**G**) HEK-Blue2™ cells (CD14^+^ cells) and HEK-TLR2 cells (no CD14 cells) were activated with LTA 1μg/ml or Pam_3_CSK_4_ 100ng/ml. The presence of CD14 and activation of IκB (NF-κB) were analyzed by Western blot. CD14 controls NF-κB activation in TLR2 ligands-activated cells. Data are representative of 3 independent experiments.

Previous studies indicate that LTA [14] and Pam_3_CSK_4_ [7,10] interact with CD14 and that the internalization of TLR4 is controlled by CD14 [5]. Using the loss of cell surface expression and intracellular accumulation of LTA and Pam_3_CSK_4_ as a read-out for efficient endocytosis, we show that CD14 controls LTA and Pam_3_CSK_4_ internalization. While the amount of LTA and Pam_3_CSK_4_ bound to on the cell surface of CD14 negative cells is the same that of CD14 positive cells (Figures S3A and S3B), internalization of TLR2 ligands depends of the expression of CD14 on the surface of HEK-Blue2™ cells (Figure 6A-D). In agreement with Figures 1, 3 and 4 indicating that endocytosis is important for TNF expression induced by LTA and Pam_3_CSK_4_, CD14 negative (CD14^-^) cells produce a lower level of IL-8 compared to CD14 positive (CD14^+^) cells in response to LTA and Pam_3_CSK_4_ (Figure 6E). Moreover, specific monoclonal blocking Abs against CD14 reduces the TNF responses in LTA- and Pam_3_CSK_4_-activated monocytes (Figure 6F). In further experiments, IκB was degraded in CD14^+^ cells, but not in CD14^-^ cells upon stimulation with LTA and Pam_3_CSK_4_ (Figure 6G). Interestingly, responses were markedly reduced, but not completely absent, in CD14 knockout cells or in monocytes treated with blocking antibodies (Figure 6E and 6F). This effect could not be explain by the participation of the scavenger receptors CD36, that was show to participate to TLR2 signaling, because CD36 is not expressed by CD14^+^ or CD14^-^ cells (Figure S3C).

Altogether, these results demonstrate that CD14 controls TLR2 internalization and facilitates NF-κB activation and resulting TNF expression in response to LTA and Pam_3_CSK_4_.

## Discussion

TLRs are classified according to the ligands they recognize and their cellular location. Due to the cell surface localization of TLR4 and TLR2, it was originally assumed that cell signaling from these receptors took place from the cell surface of many cell types. However, recent studies have challenged this assumption by demonstrating that LPS binding by cell-surface expressed TLR4 was able to induce a specific signaling pathway from an endolysosomal compartment [2]. Here, we demonstrate that TLR2, together with its co-receptors, TLR1 and TLR6, requires internalization to trigger NF-κB activation in response to LTA and Pam_3_CSK_4_, providing a novel understanding on how TLRs coordinate ligand recognition and subsequent triggering of a specific signalization. In two separate experimental systems, i.e. primary human monocytes and HEK-Blue2™ cells, our data further indicate that clathrin-dependent endocytosis mediates NF-κB activation, and that this mechanism is controlled by CD14.

The effect of internalization on TLR4 signaling has been well characterized. While TLR4 induces early NF-κB activation from the cell surface through TIRAP/MyD88-dependent pathways, a TRAM/TRIF-dependent pathway triggers late NF-κB activation and IFN-β expression from endosomal compartments [2]. Although proinflammatory cytokine production depends on both signaling from the cell surface and endosomal compartments, several lines of evidence suggest that the endosomal-dependent signaling pathway is more important. In particular, recent advances have demonstrated that peritoneal macrophages derived from TRAM^-/-^ or TRIF^-/-^ mice produce neither TNF nor IL-6 in response to high dose of LPS [25], and that, pharmacological inhibition of TRAM and endocytosis impaired NF-κB activation and IL-6 production in LPS-treated cells [2,26]. With regard to TLR2, while numerous studies have identified the presence of TLR2 in endosomal compartments [3,13,14], only few studies have investigated the functional implication of endocytosis in TLR2 signaling [11,24]. Of specific importance, TLR2-TLR1 and TLR2-TLR6, which signal through TIRAP and MyD88, are expressed on the cell surface and are recruited to the phagosome to induce IFN-β expression in monocytes upon viral infection [3] However, it remains unknown whether the cellular localization of TLR2 is important for NF-κB activation induced by LTA and Pam_3_CSK_4_ in monocytes. While TLR2 internalization, via a CD36-dependent mechanism, was reported to be required for TNF expression, solid data demonstrating an effect of this mechanism on NF-κB activation are still missing [27,28]. Supportive insights were however provided by the following data: (i) FSL-1, a TLR2 and TLR6 ligand analogue to LTA, is taken up by murine macrophage cell lines [18], (ii) the signaling triggered by Pam_3_CSK_4_ (TLR2 and TLR1 ligand) was detected from endosomal compartments although the pathways involved were not defined [15], (iii) *Staphylococcus aureus*, from which LTA is purified, induced pro-inflammatory cytokines only after internalization [27], and (iv) it was reported that a TLR2-P631H mutant (TLR2-P631H is a single nucleotide polymorphism) affects the rate of internalization of the wild type TLR2 and NF-κB activation in a dominant negative fashion [29]. 

Localization of TLR2 into endosomal vesicles raises the question of the nature/identity of the pathway used for its internalization. Thus, our results suggest that the clathrin/dynamin-dependent endocytic pathway (Figures 2 and 3A) is involved in TLR2 internalization and NF-κB activation. While TLR2 is reported to be associated to lipid rafts [14,30], their disruption does not significantly affect LTA or FSL-1 internalization [15,18]. However, one could also assume that both specific pathways might be involved to fulfill different functions, i.e. the lipid raft pathway for passive recycling of receptors and the clathrin/dynamin pathway for active endocytosis-dependent signaling.

While our data indicate (Figure 4A) that both free LPS and LPS-coated beads are capable of activating NF-κB, only free LTA and Pam_3_CSK_4_, but not LTA- and Pam_3_CSK_4_-coated beads could activate NF-κB. These results are consistent with data indicating the inability of Pam_3_CSK_4_ to induce IL-6 secretion from U937 macrophages when immobilized on a cell culture plate [15] and the observation that internalization is critical for TLR2-mediated recognition of LTA [19]. Similar to the requirement of NF-κB in driving TNF and IL-6 expression in LPS-activated monocytes [22,23], our results and results from other groups show that endocytosis inhibition decreases TNF and IL-6 expression induced by TLR2 ligands in primary human monocytes (Figure 1A-1B), RAW264.7 cells [24] and U937 cells, respectively [15]. Taken together these results demonstrate that endosomal localization of TLR2 is important for NF-κB activation. 

CD14 has been described as an important TLR4 co-receptor in LPS recognition and was shown to control TLR4 internalization and type I IFN expression [5,31]. While CD14 was also found to serve as a co-receptor for the recognition of TLR2 ligands [7,10,11], its role in NF-κB activation triggered by TLR2 ligands remained to be established. Here, we further demonstrate that LTA and Pam_3_CSK_4_ internalization is CD14-dependent. This suggests a comparable role for CD14 in cell activation by both TLR4 and TLR2 ligands. Interestingly, other evidence also indicates that CD14 is required for DNA uptake and delivery to TLR7 and TLR9 [32] and that CD14 knockdown decreased FSL-1 internalization in macrophages [18]. In support of a functional implication of endocytosis in NF-κB activation downstream TLR2 signaling (Figures 2 and 3A), our results demonstrate that CD14-dependent internalization of LTA and Pam_3_CSK_4_ regulates NF-κB activation (Figures 6A-D and 6G). Our data, however, indicate that CD14 may only be partially required for TLR2-mediated TNF and IL-8 expression (Figure 6E and 6F) or enhance the efficacy of ligand TLR2 interaction as stated below and that other mechanisms are likely to be required to generate a maximal response. Possibly, CD36 could participate to such additional mechanisms. However, whereas CD36 was shown to increases the response to LTA in HEK293 transfected cells, it was not essential [6]. Furthermore, CD36 is not required for cell activation by Pam_3_CSK_4_ [6,33]. Thus, CD36 seems to be implicated in some aspects of TLR2 activity dependent on the ligand and the cell type. Thus, HEK293 Blue cells (CD14^+^) and HEK293 TLR2 cells (CD14^-^) do not express CD36, but respond to LTA and Pam_3_CSK_4_ (Figures 6 and S3). As cellular responses of HEK cells are dependent on ligand internalization, we conclude is not required for endocytosis of LTA and Pam_3_CSK_4_.

It is also possible that CD14 is required for early uptake of ligands at low concentration by clathrin-dependent endocytosis, while other regulators may contribute to late uptake of ligands at higher concentrations. In support of this hypothesis, it was shown that TLR2 signaling may vary depending on ligand concentration [34] and that CD14 is required for recognition of low concentrations of LPS [35]. Furthermore, Zanoni et al. have shown that while CD14 is important for TLR4-MyD88-dependent signal transduction only at low concentrations of LPS, the function of CD14 is independent of the signaling triggered by TLR4 obtained at high LPS concentrations [5]. Likewise, while MyD88 was shown to be critical for TNF production in macrophages in response to *S.Aureus*, LTA and Pam_3_CSK_4_, it did not play a significant role in pathogen internalization, indicating that TLR2-mediated endocytosis and selective signaling are functionally separated (Figure 5C) [27].

In conclusion, our study reveals that NF-κB activation by LTA and Pam_3_CSK_4_ is regulated via a clathrin- and CD14-dependent endocytosis of TLR2 into primary human monocytes. Thus, although present at the plasma membrane, TLR2 ligands induce NF-κB activation from endosomal compartment by a MyD88-dependent mechanism.

## Supporting Information

Figure S1 **Effects of weak base NH_4_Cl and Endosomal inhibitors on TNF secretion and viability of human monocytes.** Related to Figures 1 and 2. (**A**-**B**) Dose response of the effect of weak base NH_4_Cl on TNF secretion in TLR2 ligand-activated monocytes. The TNF response to LTA and Pam_3_CSK_4_ after 24h was strongly reduced by NH_4_Cl. Data are represented as mean +/- SD of 3 independent experiments.
(**C**) Viability of human monocytes at 4h and 24h and Viability of HEK-Blue2™ cells at 24h treated with highest endocytosis inhibitor concentration used in the experiments, as assessed by Trypan blue exclusion. Data are represented as mean +/- SD of 2 independent experiments.
(TIF)

Figure S2 **Blocking peptide effects on Poly I:C-induced TNF production.** Related to Figure 5. Human monocytes were treated with blocking peptides for MyD88 and TRIF or control peptide (CP) during 60min prior to be activated for 24h with Poly I:C (10μg/ml). TRIF blocking peptide reduces TNF response to Poly I:C after 24h. Data are represented as mean +/- SD of results obtained with monocytes from at least 3 different blood donors.
(TIF)

Figure S3 **Expression and quantification of TLR2 ligands and TLR2 coreceptors on cell surface.** Related to Figure 6. (**A**) Representative experiment of the presence of LTA-biotin and Pam3CSK4-biotin on HEK-Blue2™ (CD14^+^) cells and HEK-TLR2 cells (CD14^-^) cells surface.
(**B**) HEK-Blue2™ cells and HEK-TLR2 cells were treated with LTA-biotin 1μg/ml or Pam_3_CSK_4_-biotin 100ng/ml on ice and their presence on cells surface was quantified by flow cytometry. Displayed are the mean +/- SD of MFIs of specific cell surface TLR2 ligands staining of at least 3 independent experiments.
(**C**) Cells surface expression of CD36 and CD14 in human monocytes, HEK-Blue2™ (CD14^+^) cells and HEK-TLR2 (CD14^-^) cells. Data are representative of 3 independent experiments.
(TIF)
