# Supplementary figures and images for: TLR2 Ligands Induce NF-κB Activation from Endosomal Compartments of Human Monocytes
Source: PLoS One. 2013 Dec 12;8(12):e80743. doi: 10.1371/journal.pone.0080743 (PMC3861177; doi:10.1371/journal.pone.0080743)

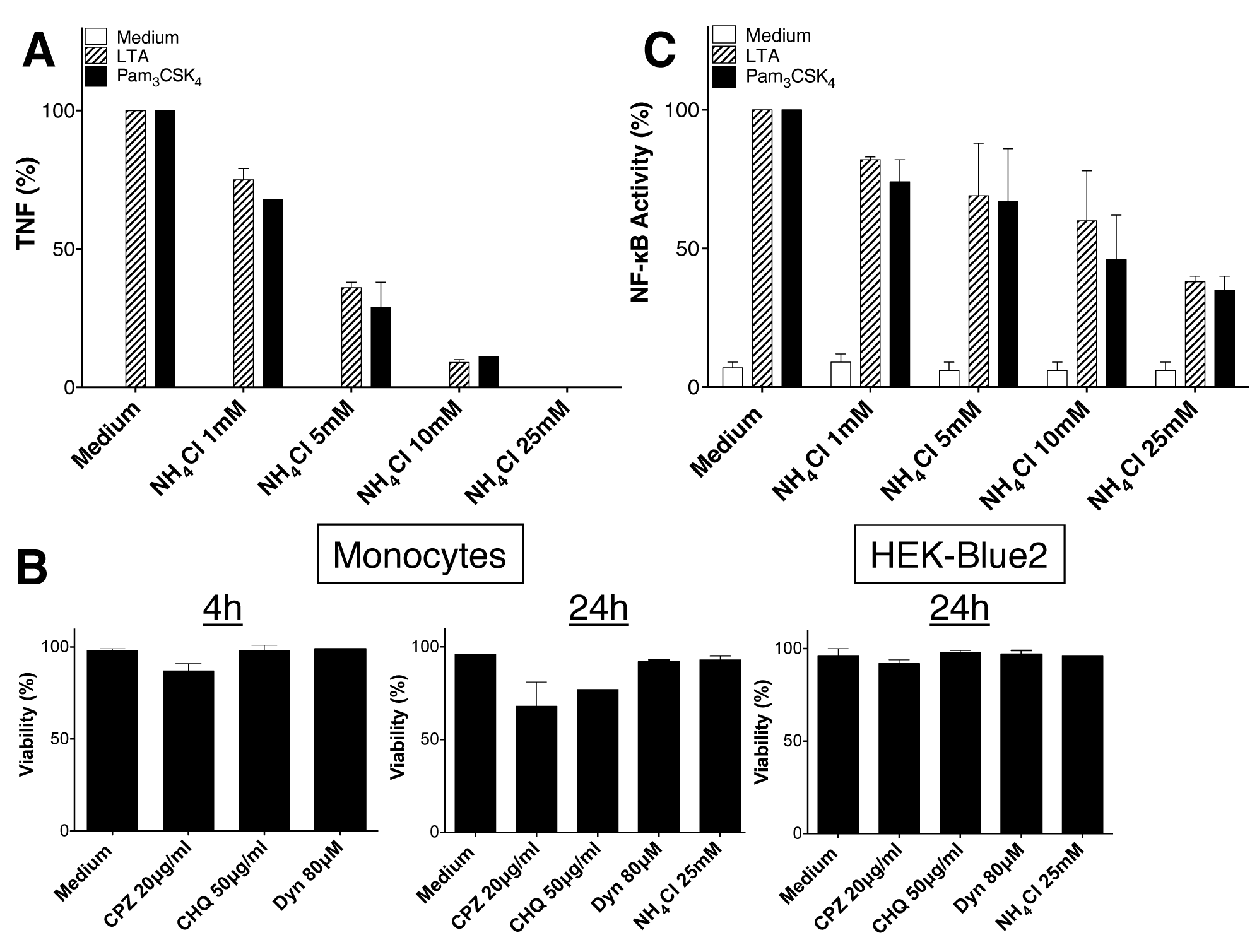

Supplement: Figure S1 — Effects of weak base NH4Cl and Endosomal inhibitors on TNF secretion and viability of human monocytes. Related to Figures 1 and 2. (A-B) Dose response of the effect of weak base NH4Cl on TNF secretion in TLR2 ligand-activated monocytes. The TNF response to LTA and Pam3CSK4 after 24h was strongly reduced by NH4Cl. Data are represented as mean +/- SD of 3 independent experiments. (C) Viability of human monocytes at 4h and 24h and Viability of HEK-Blue2™ cells at 24h treated with highest endocytosis inhibitor concentration used in the experiments, as assessed by Trypan blue exclusion. Data are represented as mean +/- SD of 2 independent experiments. (TIF) [file pone.0080743.s001.tif]

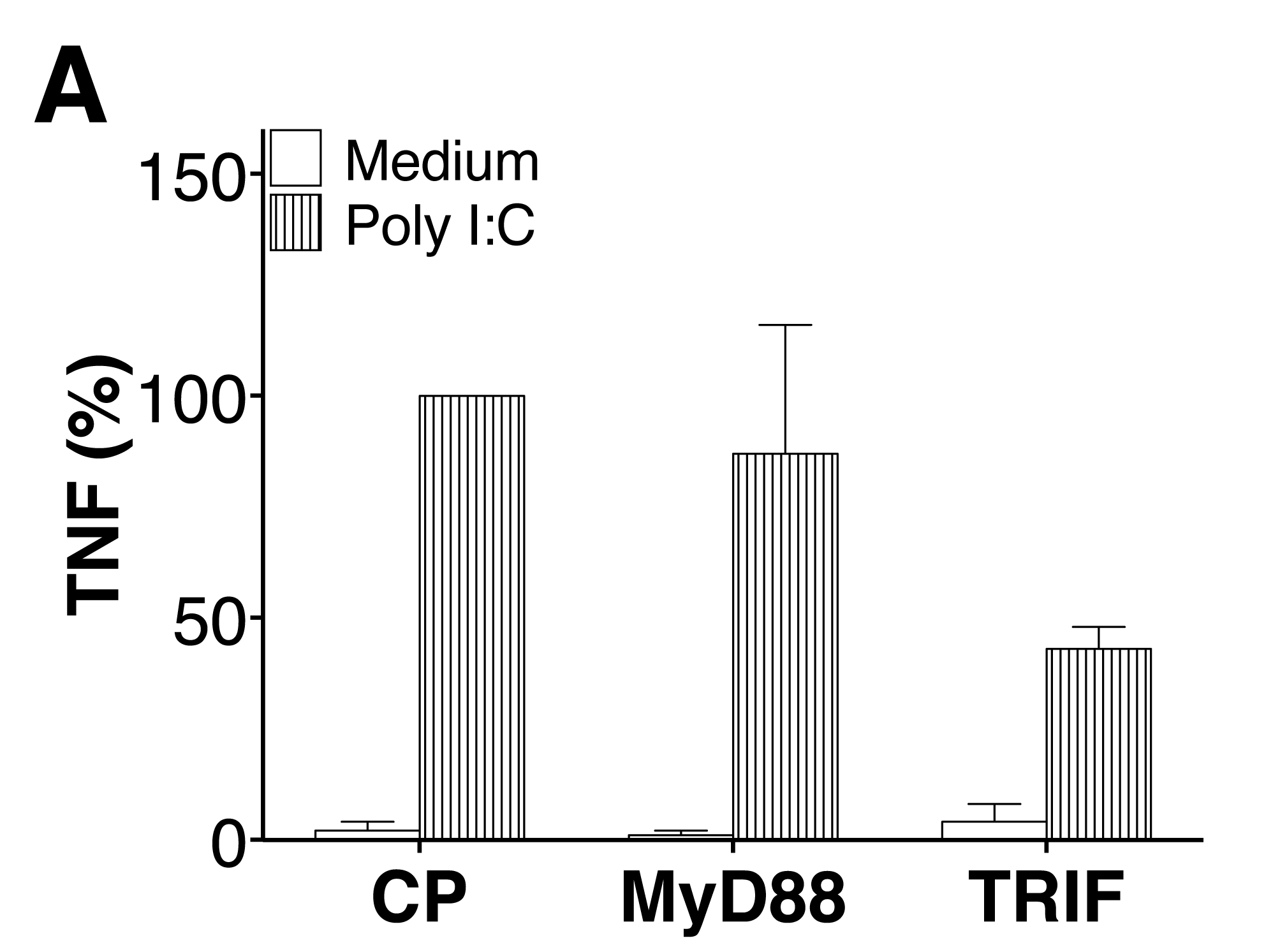

Supplement: Figure S2 — Blocking peptide effects on Poly I:C-induced TNF production. Related to Figure 5. Human monocytes were treated with blocking peptides for MyD88 and TRIF or control peptide (CP) during 60min prior to be activated for 24h with Poly I:C (10μg/ml). TRIF blocking peptide reduces TNF response to Poly I:C after 24h. Data are represented as mean +/- SD of results obtained with monocytes from at least 3 different blood donors. (TIF) [file pone.0080743.s002.tif]

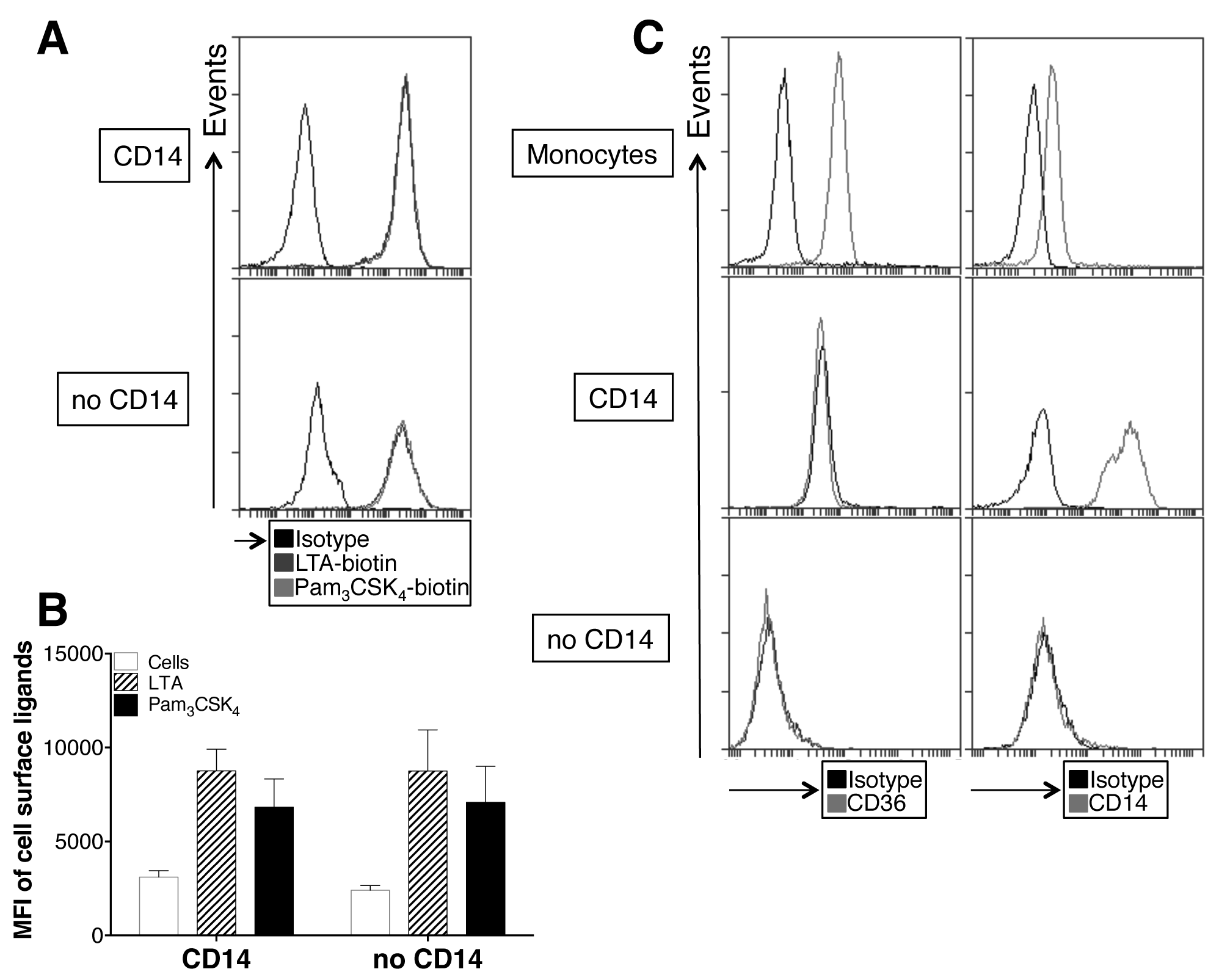

Supplement: Figure S3 — Expression and quantification of TLR2 ligands and TLR2 coreceptors on cell surface. Related to Figure 6. (A) Representative experiment of the presence of LTA-biotin and Pam3CSK4-biotin on HEK-Blue2™ (CD14+) cells and HEK-TLR2 cells (CD14-) cells surface. (B) HEK-Blue2™ cells and HEK-TLR2 cells were treated with LTA-biotin 1μg/ml or Pam3CSK4-biotin 100ng/ml on ice and their presence on cells surface was quantified by flow cytometry. Displayed are the mean +/- SD of MFIs of specific cell surface TLR2 ligands staining of at least 3 independent experiments. (C) Cells surface expression of CD36 and CD14 in human monocytes, HEK-Blue2™ (CD14+) cells and HEK-TLR2 (CD14-) cells. Data are representative of 3 independent experiments. (TIF) [file pone.0080743.s003.tif]
